# Artistic Freedom or Animal Cruelty? Contemporary Visual Art Practice That Involves Live and Deceased Animals

**DOI:** 10.3390/ani11030812

**Published:** 2021-03-14

**Authors:** Ellie Coleman, Rebecca Scollen, Beata Batorowicz, David Akenson

**Affiliations:** School of Creative Arts, Faculty of Business, Education, Law and Arts, University of Southern Queensland, Darling Heights, QLD 4350, Australia; ellie.frey@usq.edu.au (E.C.); beata.batorowicz@usq.edu.au (B.B.); david.akenson@usq.edu.au (D.A.)

**Keywords:** animals, visual art, ethics, cruelty, human–animal hierarchy, animal rights

## Abstract

**Simple Summary:**

This paper examines a selection of 21st-century international examples of exhibited visual artworks involving live or deceased animals. It seeks to reveal the risks and benefits of unique encounters with animals through art and to consider the ethical implications of artwork deploying animals. Australian and international animal protection laws are not explicit when it comes to the sourcing of animals for art nor for the direct inclusion of animals in artworks. This lack leads to a variety of artistic practices; some considered ethical while others are viewed as controversial, bordering on animal cruelty. Recommendations of how to better determine what is the acceptable use of animals in art with a view to informing legal guidelines and artistic best practice are presented.

**Abstract:**

This paper examines a selection of 21st-century international examples of exhibited visual artworks involving live or deceased animals. It seeks to reveal the risks and benefits of unique encounters with animals through art and to consider the ethical implications of artwork deploying animals. Australian and international animal protection laws are not explicit when it comes to the sourcing of animals for art nor for the direct inclusion of animals in artworks. This lack leads to a variety of artistic practices, some considered ethical while others are viewed as controversial, bordering on animal cruelty. Artwork selection is determined by a focus on high-profile artists who intentionally use animals in their practice and whose reputation has been fostered by this intention. The study provides insight into how the intentional use of ethically sourced animals within art practice can be a method of addressing hierarchal human–animal imbalances. Further, this study identifies unethical practices that may be best avoided regardless of the pro-animal political statements the artists put forward. Recommendations of how to better determine what is an acceptable use of animals in art with a view to informing legal guidelines and artistic best practice are presented.

## 1. Introduction

Animals have been depicted in visual art for thousands of years; whether it be via cave paintings and ceremonial costumes made by ancient indigenous communities, or via representations on canvas as part of an idyllic rural scene, busy marketplace, or the family hearth. Animals have always been a part of the human landscape and, as such, they have featured in art as a method of our shared communication and meaning making. However, in more recent times, contemporary art has taken the incorporation of animals in artworks a step further. Some contemporary artists choose to directly include animals as part of the artwork to make political statements about our relationships with animals and what that can signify in society. This method of using animals in art practice involves an experiential and tactile encounter that expands our understandings of what art is and the role it plays in everyday life.

The late 20th-century German artist Joseph Beuys was renowned for blurring art and life by using live and deceased animals in his art performances. Beuys approached his performances with animals as a means of expanding traditional notions of art, ultimately becoming part of a broader social consciousness through what he referred to as “Social Sculpture” [1] (p. 41). Beuys’ Social Sculpture theory is concerned with art being integrated with everyday life as he felt everyday life encompasses creativity and creative action. In this sense, Beuys argued that everyone could be an artist [1]. Beuys’ *I Like America and America Likes Me* (1974) [1] is a pivotal work where the artist takes on a quasi-shaman or shepherd persona and is filmed during his three days locked in a cage with a wild coyote. The varying degree of interaction between the shaman and coyote across the three-day period is symbolic of German and American World War Two tensions as well as broader dichotomies between themes of being wild versus civil and free versus captive in the search for healing [2]. There are claims that actions such as these, and many other artworks since, also assist to raise awareness of the plight of animals and to reveal human–animal relationships. By including real animals in the artworks, there is an integration of art and everyday life which can see the works as effective tools of communication to point out how our treatment and consumption of animals needs closer examination. Yet, the methods in which animals are included in some of the works are contentious, eliciting the question: are these examples of artistic freedom (works created free of censorship and ethical considerations) framed by an animal welfare agenda, or simply cases of *animal cruelty* (unnecessary, unreasonable or unjustifiable pain, harm or suffering to an animal) produced and exhibited in the name of art—a practice that serves to reinforce humanity’s subjugation of animals?

This study examines a selection of 21st-century international examples of exhibited artworks containing animals. Selection is determined by a focus on high-profile artists who intentionally use animals in their practice and whose reputation has been fostered by this intention. The works have also generated public comment about the ethics of making and exhibiting artworks of this nature. A review of this kind seeks to capture a public debate within an academic enquiry to reveal unique encounters with animals through art and to consider the ethical implications of artwork involving animals, whether alive or deceased. In doing so, the study seeks to offer insight into how the intentional use of ethically sourced animals within art practice can provide a method of addressing hierarchal human–animal imbalances. Here, the “ethical sourcing” of animals within art refers to animals and animal remains that were obtained in the most humane ways possible [3] and were not killed specifically to be included in the artworks [4], nor mistreated in the name of art. Further, this study seeks to identify both ethical, and most importantly, unethical practices that are best avoided regardless of the pro-animal political statements the artists contend they put forward.

## 2. Perception and Representation of Animals

### 2.1. Human–Animal Hierarchy and Animal Rights

The perception of animals is often dependent on their seeming utility to humans [5]. Judgements of utility are socially constructed ideologies that are deeply rooted within culture [6]. These ideologies gained traction within western society through the works of Aristotle (ca. 322 BCE) who presented the notion of Scala Naturae [7]—a human–animal hierarchy and further developed by LoveJoy’s *The Great Chain of Being: The History of an Idea* (1936) [8]. Aristotle and Lovejoy deny that animals warrant moral status due to philosophical theories of the nature of the world and the proper place of its inhabitants [9]. Moral status is a concept that determines who, or what, is valuable enough that they have their interests considered [10]. This understanding of animals in relation to humans led to the later concept of Speciesism [11], further defined here by Gary Yourofsky [12] as, “The unethical, unprincipled point of view, that the human species has every right to exploit, enslave and murder another species because we believe that our species is so more special, so more superior than the other ones, that we’re the only ones that count, and we’re the only ones that matter.” Morton et al. [13] argue that a form of speciesism appears to be at play when it comes to current Australian animal welfare legislation where the pain and suffering of companion animals is taken more seriously than that produced by humans against farm or feral animals. Yet, recent acceptance and inclusion of animal sentience in animal welfare legislation in some states of Australia should, in theory, counteract speciesism and ensure all species are protected.

The concept of animal sentience has been accepted within the scientific community since the early 19th century [14]. Animal sentience is the capacity of an animal to experience a variety of feelings such as suffering, boredom, fear, and joy. Sentience also extends to an animal’s ability to learn from experience and from other animals, to assess risks and benefits, and to make choices [14]. This belief was endorsed by William Youatt (1839) [15] who suggested that animals have senses, emotions and consciousness. He argued that they demonstrate sagacity, docility, memory, association of ideas and reason [15]. Youatt insisted they also have imagination and the moral qualities of courage, friendship and loyalty [14] (p. 11). Australian researchers contend that when it comes to animal protection laws “it has been claimed by some scholars that an animal’s capacity to experience both negative and positive emotions has been the driving factor for the animal welfare movement and consequential animal protection legislation. Thus, it is notable that many laws neglect to acknowledge or discuss animal sentience when defining ‘animals’” [13] (p. 14). The Australian Capital Territory (ACT) first acknowledged sentience in its legislation via the 2019 amendment of the Animal Welfare Act 1992 to include the following statement:

“The main objects of this Act are to recognize that—(a) animals are sentient beings that are able to subjectively feel and perceive the world around them; and (b) animals have intrinsic value and deserve to be treated with compassion and have a quality of life that reflects their intrinsic value; and (c) people have a duty to care for the physical and mental welfare of animals”.

[13] (p. 15)

Now more recently, the Australian state of Victoria has amended its legislation due to “science telling us that animals are sentient” [13] (p. 15). This places Australia more in line with the European Union who included the notion of sentience in the Treaty of the Functioning of the European Union in 2012 [16].

Peter Singer [17], Gary Francione [18], and Maciej Henneberg [19] also argue for the importance of acknowledging animal intelligence. Human–animal hierarchy makes a judgement on an animal’s intelligence based on human ability to understand, whereas animal intelligence must be perceived and understood in its own context, or on its own terms rather than from a human perspective [20]. This premise addresses the idea that it is inappropriate to judge an animal on an ability which they do not possess and arguably do not need to possess [21]. This too exposes the lack of understanding or consideration of the various forms that intelligence can take place, and that this intelligence differs from, rather than is lesser to, the human experience.

The influence of human–animal hierarchical models is still evident within some parts of our society. However, the acknowledgment of animal sentience fueled the opposing argument for the respectful and ethical treatment of animals [22,23]. Jeremy Bentham (1789) wrote, “The question is not, can they reason? Nor, can they talk? But, can they suffer?” [24] (p. 640). Peter Singer later adopted this reasoning in *Animal Liberation* (1975) [11]. Singer’s philosophy is frequently credited for the creation of the animal rights movement with a focus on recognizing animal suffering [25]. Francione [17] furthers the argument stating veganism as a moral imperative for animal rights. If animals matter morally, he claims we are morally obligated to stop eating, wearing, and exploiting them. The view that an animal’s moral status is equivalent to a moral right means any action that fails to treat the animal as a being with inherent worth would violate that animal’s rights and is therefore morally objectionable [12]. Regan [26] (p. 24) states:

“…animals are treated routinely, systematically as if their value were reducible to their usefulness to others, they are routinely, systematically treated with a lack of respect, and thus are their rights routinely, systematically violated.”

Animal rights are therefore based on the philosophical position that discrimination between humans and animals, on the basis of species, is arbitrary and unacceptable [27] (p. 75). According to Francione [18], animal rights involve a rejection of all animal use in everyday living, no matter how “humane” the treatment of animals is because of the underlying premise that animals have interest in continuing to live. In doing so, animal rights philosophy is underpinned by the central idea that animals are not ours to exploit for food, clothing, entertainment, or experimentation [18]. As such, Francione disagrees with the use of animals in art stating “I do not believe in using animals for such purposes and every instance of such use that I have seen is nothing but outright exploitation” [28] (p. 8). This positioning was advocated by Regan [26] (p. 179) who believes the fundamental wrong is the system which allows us to view animals as our resources, here for us to be eaten, surgically manipulated, or exploited for sport or money. Regan established the argument that value belongs equally to those who are the experiencing subjects of life [29]. This refers to beings who have subjective awareness of their own lives as well as desires, memories, feelings, emotions, self-consciousness, and a sense of their own future [29]. In consideration of this, if animals matter morally then they must have at least one right; the right not to be used as a resource.

Animal protection laws vary from country to country [30], and in places like Australia, where the authors of this paper reside, they vary from state to state [13]. “Some of the criticisms of a state-based approach in Australia and elsewhere include that it makes national data collection almost impossible, causes public confusion, does not allow for cross-jurisdictional recognition of animal prohibition orders and does not present a united front toward animal protection” [30] (p. 2). These inconsistencies further cloud the determination of appropriate treatment of animals and the methods of their inclusion in artwork. The Australian Animal Care and Protection Act (2001) [31] fails to outline the parameters of incorporating animals (live or deceased) in contemporary art or art research. Consequently, there are no set policies in this field, with artists left to follow the country’s animal protection laws in the State which their work is exhibited [32].

Queensland is the only state in Australia with an Exhibited Animals Act (2015) [33] which outlines the conditions of when and how live animals can be lawfully exhibited. According to the Act “to exhibit an animal means to display the animal to the public, including, for example, for commercial, cultural, educational, entertainment or scientific purposes” [33] (pp. 18–19). Venues for display can include such places as agricultural shows, circuses and zoos, animal fancier association meetings, and places of education [33] (pp. 18–19). There is no mention of art galleries within the Act, but arguably, by extension artists operating in this State can take note in the Act that an animal can be kept on display for up to eleven days at a time if the responsible person meets certain legal conditions [33] (p. 17). Guidelines to protect animals when exhibited can also be found in international legislation such as the Animal Welfare (Licensing of Activities Involving Animals) (England) Regulations 2018 [34], and the United States Department of Agriculture Animal Welfare Act and Animal Welfare Regulations 2020 [35].

Acts such as the Exhibited Animals Act (2015) are designed to protect the welfare of animals and to penalize those who are found guilty of animal cruelty. Most states in Australia define cruelty “as causing an animal unnecessary, unreasonable or unjustifiable pain, harm or suffering, where pain, harm or suffering is defined as forms of distress and injury to animals” [13] (p. 7). This is in keeping with international legislation such as the Swiss Animal Welfare Act (2017) which stipulates that “No one shall unjustifiably expose animals to pain, suffering, physical injury or fear” [36]. In countries such as Australia and the United Kingdom [37], responsibility is placed on the “person in charge” or the “owner” of an animal when it comes to maintaining an animal’s welfare and protection [13]. When it comes to penalties, “it is the person who has ‘custody and control’ of the animal at the point of time of an offence” [13] (p. 18) who will be charged. This has implications for artists who involve live animals in their artworks, as under this Act they are responsible for the animals’ welfare and protection.

### 2.2. Animals in Contemporary Visual Art—Ethical or Unethical?

Framed by these ethical debates is this study’s exploration of how animals are employed within contemporary art in the 21st century, what is considered ethical and unethical use of animals within art practice, and more specifically, to identify the conflicts between artistic freedom and animal rights. Artistic freedom refers to the freedom to imagine, create and distribute diverse cultural expressions free of governmental censorship, political interference or the pressures of non-state actors [38]. Artistic freedom regarding the use of animals in contemporary art can mean the artist has limited or no ethical considerations for the animals involved [39]. Baker [40] (p. 70) discusses how the move toward physically using animals in artwork has been seen as both “ethically and aesthetically disturbing”. Understanding the different ways that animals are employed and exploited within contemporary art is important in terms of challenging the notion of animals being devalued.

Recognising that animals are unethically used within art practice is critical to this study as it highlights the lack of ethical considerations within contemporary art as well as assists in establishing boundaries on what is acceptable in terms of animal ethics. Other forms of cultural endeavour and entertainments that feature animals, such as circuses, marine parks, and zoos have come under increased public pressure in the 21st century for holding animals captive and for making them “perform” for human amusement [41] (p. 5). If the involvement of animals in these venues is questioned, what is our societal stance on the use of live and deceased animals in visual art contexts? For example, in Beuys’ previously mentioned work *I Like America and America Likes Me* (1974) it can be argued that the wild coyote is held captive in an art gallery and, therefore, becomes part of the spectacle for the sake of an art performance [42].

As there is ethical and legal scope yet to be covered in the use of animals in art, it is important to acknowledge that there has been much discussion pertaining to the use of human bodies in contemporary art that have included ethical, medical and legal perspectives [43]. This is exemplified through artists such as the American-based Andrew Krasnow who used human skin in art as a commentary on America’s ethics regarding war and human cruelty [44], as well as the German anatomist Gunther von Hagens’ whose renowned *Body Worlds* exhibition consisted of full plasticized human corpses posed for their educational potential [45]. von Hagens invented plastination, the method of replacing water and fat in human tissue with certain plastics, preserving them for study [46]. The exhibition is regarded as controversial as the work’s “educational aims are ambiguous, and some aspects of the exhibit violate human dignity” [45] (p. 12). The multidisciplinary emphasis on the ethical issue of using human bodies over animal remains in exhibitions such as *Body Worlds*, offers a key counterpoint while reinforcing this paper’s broader query as to why more extensive guidelines for best ethical and lawful practices in the use of animals in art have not yet been fully developed. Potentially, this also reiterates the argument of the ongoing human–animal hierarchal imbalances permeating contemporary society.

Underpinning this investigation is the work of seminal contemporary artists such Julia deVille (Australian) and Angela Singer (British/New Zealander) who employ, we will contend, ethically sourced animals in their art practice. The study also explores the work of artists who arguably contribute to the unethical treatment of animals within art practice [23] such as Adel Abdessemed (Algerian-French), Herman Nitsch (Austrian), Wim Delvoye (American), and Guillermo “Habacuc” Vargas (Costa Rican). These divergent approaches to the use of animals in art practice reflect the complexity of animal rights within contemporary art. Yet, there is limited discourse surrounding the perceived ethical and unethical uses of animals in art practice [42]. As art historian, Steve Baker [40] (p. 70) states: “In terms of where artists choose to set their limits, there are some genuinely complex cases where the artist is clearly working with seriousness, awareness, and a sense of integrity, but where I’m personally uncomfortable with some of their decisions and actions.”

#### 2.2.1. Vargas

Guillermo Vargas’ installation titled *Exposición No.1*. (2007) [47] presents a live, emaciated street dog tied up without food or water in a gallery. Accompanying the dog is the statement, “Eres lo que lees,” meaning “You are what you read” written on the gallery wall with dog biscuits [47]. Due to public outrage over the exhibit and not knowing whether this dog starved to death or survived, a petition was distributed calling for Vargas to be excluded from the 2008 *Bienal Centroamericana en Honduras* where he planned to recreate the work [48].

Though this installation caused controversy, Vargas highlighted the lack of ethical guidelines for artists and demonstrated the hypocrisy of societal response. That is, by placing a street dog in a gallery setting suddenly his or her predicament becomes an “ethical problem”, in contrast to many stray animals suffering unnoticed on the streets [47]. In this way “objectifying” and “devaluing” the dog within an art context elicits public responses which aid in the process of identifying what is ethical or unethical treatment of animals. This work demonstrates that the gallery, or any art space for that matter, can act as a platform for animal welfare issues to be raised and engaged with. It also raises questions though concerning artistic credibility, animal rights, and the morality of contemporary art which requires critical exploration and analysis [49]. The public disagreement with the work clearly highlights that people view this installation as unethical [28]. It is uncertain how many days the dog was tied up in the gallery as Vargas refused to comment on the fate the dog [50]. A Costa Rican newspaper *La Nación* [51] reported that the dog died after the first day of the show due to starvation. However, gallery director Juanita Bermudez insisted that the dog was fed, did not die, and had escaped immediately after the exhibition ended: “It was untied all the time except for the three hours the exhibition lasted and it was fed regularly with dog food” [51]. Guidelines such as those in the Queensland Exhibited Animals Act [33] would have provided a framework for how the dog was to be treated while on display, who was responsible for his/her welfare, and what steps should be taken if the animal escapes.

#### 2.2.2. Nitsch

More recently, Herman Nitsch was publicly scrutinised for his Museum of Old and New Art Dark Mofo exhibition where he had a bull slaughtered to use as a prop in a performance piece titled *150.Action* (2017) [52]. Prior to the performance the bull was killed at a local slaughterhouse, which adhered to approved legal standards. The animal’s body was then used as a prop within the performance to explore themes of ritual and sacrifice [52]. Human performers rubbed their bodies in the deceased animal’s blood and entrails during the exhibition. Nitsch’s performances involve blood, animal entrails and nudity with the intention to “stir up the audience, the participants of his performances to bring them an understanding of their existence” [53]. Dark Mofo’s creative director Leigh Carmichael [54] (p. 1) stated:

“We understand that his work will be confronting and difficult, but we will not shy away from presenting work that challenges us to consider the ethical implications of our actions both today, and in the past.”

Carmichael’s response raises a critical point in terms of how important it is to reflect upon, and question, animal ethics in relation to human actions towards animals. In particular, is it less ethical to slaughter a bull in the name of art than to slaughter it for food? This question highlights the inconsistent moral considerations given to animals when they are used for human consumption versus employed within contemporary art [55]. The public outcry engendered from this exhibition demonstrates that our culture is more comfortable with the notion of animals being killed for consumption than killed in the name of art as sentient beings whom we have some moral obligation to. It is this incongruity in reaction that David Walsh, owner of the Museum of Old and New Art (MONA), says “he hopes the piece will challenge” [54]. Yet, some Australian artists, such as Yvette Watt who, incidentally, does not use animals in her practice, publicly expressed her disapproval of Nitsch’s work stating, “150.Action is not a good work… On an ethical basis, I don’t think any animal should have to die or suffer in the name of art” [55] (cited in Walsh, 2017). Furthermore, the utilization of a slaughtered bull as a “man-handled” prop in this performance could demonstrate a lack of respect for the animal in death, denying dignity for the deceased. There is no mention of “dignity” in Australian animal welfare legislation, but the Swiss Animal Welfare Act (2017) [36] does provide guidelines as to how to ensure dignity for living animals. Dignity in death is not discussed. As such, it is evident that artists are not provided with ethical frameworks to guide their decisions and actions when it comes to appropriate engagement with deceased animals in their artworks.

#### 2.2.3. Abdessemed

Adel Abdessemed’s artwork *Don’t Trust Me* (2008) [56] stretches the definition of artistic freedom. Here, a series of looped videos of animals being bludgeoned to death in Mexico is on display. The animals killed included a pig, goat, deer, ox, horse and sheep [56]. Animal welfare groups declared the video clips as degrading and cruel, and accused Abdessemed of killing animals for the sake of art and artistic freedom. New Museum curator Massimiliano Gioni has said that Abdessemed is a practitioner of “asymmetrical realism” and a key representative of a general turn towards the “unfiltered, brutal and sincere” in contemporary art [57] (p. 1). By exhibiting this work, Abdessemed allows his audience to confront the brutal reality some animals face. These acts may not have legal ramifications (as he allegedly filmed the “normal” practice on a farm), however, as a society we must question if it is morally acceptable to film the act and then display the killing in the arena of the contemporary exhibiting space [58]. According to the Queensland Exhibited Animals Act (2015), a person is understood to be exhibiting an animal if “the person records the animal’s image for display to the public, whether the image is displayed when it is recorded or is intended to be displayed after it is recorded” [33] (p. 19). If this welfare act legislated in one State in Australia was applied in this instance, the artist as the “responsible person” and his work could be assessed through a legal process to see if it constituted animal cruelty. The public backlash demonstrates that the majority views this artwork as unethical, however, it also presents possible moral inconstancies if they also consume animals due to the context of these animals’ deaths. More importantly, this installation demonstrates that using animals in contemporary art can create a platform to discuss the routine killing of billions of animals for consumption, and perhaps, from that point of view, Abdessemed’s films might be considered ethical.

#### 2.2.4. Delvoye

Whilst Abdessemed films the final living moments of farm animals, Wim Delvoye has a more complex relationship with the pigs he uses in his practice. Delvoye tattooed the skins of dead pigs until 1997, when he commenced tattooing pigs while they were alive [59]. In 2004, he relocated from the United States to China to become owner and operator of a small farm called “Art Farm” to specifically raise the pigs for his work [60]. Delvoye’s artworks involve placing the pigs under a general anaesthetic and tattooing them, before they are slaughtered and skinned, with the skins themselves becoming the final artwork, either pinned flat to walls or, on some occasions, taxidermied into the form of the pig [52]. During an interview, Delvoye revealed that due to the laws regarding animal welfare being less strict in China, he was able to continue his art practices [60]. Delvoye hoped to “save” pigs from the brutality of the Chinese slaughterhouses, and after being tattooed he intended for the pigs to live through to old age on the farm. Although when questioned about the life span of the pigs, Delvoye says: “We start tattooing around 25 or 30 kilos and then we stop when he’s 100 kilos” [60] (p. 29). Delvoye [61] (p. 26) justifies his practice by saying:

“…he feels like Oscar Schindler when he visits the farm to pick out his half-dozen or so animals, experiencing guilt for those left behind, not only because they will have much shorter lives, but because they will only be valued as butchered meat, they will not bear the price tag of art.”

From an animal rights perspective a more “humane” death or the price tag of art does not excuse the killing of animals. Arguably, Delvoye’s original rescue of the pigs is beneficial to them, but ultimately, they are being exploited and slaughtered for art [28]. If the Swiss Animal Welfare Act was employed in this instance, it could be argued that the dignity of the animals has been disrespected as there is “major interference with [the animals’] appearance or abilities” [36]. Delvoye’s plan to “save” the pigs from the slaughterhouse is debatable in its efficacy; nevertheless, his work exposes complex issues surrounding the ethical use of animals in contemporary art.

#### 2.2.5. Evaristti

A further example of animal death in the name of art includes the infamous work by Evaristti, titled *Helena* (2000) [49]. This work was comprised of ten blenders, each containing a live goldfish [49]. During the exhibition, Evaristti allowed his audience to switch on the blenders if they desired, placing the ethical responsibility on them. What makes the work charged with its attractive and repulsive preoccupation is the tension it instantly creates in its viewers. By facilitating the audiences to engage or not in the action of killing a goldfish they become either the voyeuristic viewer, potential killer or the inevitable moralist whilst exposed to the work [62]. This installation, in being reliant on the audience’s decisions regarding the fate of the goldfish, was about testing and questioning ethical boundaries, particularly the individual’s moral thresholds [63]. The artwork investigates whether participants have a capacity to “kill” and, if so, how these choices provide insight into current human–animal relationships. This killing is not done out of necessity of circumstance but done for the sake of mere curiosity and for art’s sake. Therefore, the viewer that commits the killing of the goldfish does it simply out of an opportunity being presented in a gallery setting. Hofbauer [64] (p. 65) argues:

“The participatory element in this otherwise sculptural work leads to a modification of reality, in which it is not Evaristti who comes over as irresponsible, but where he passes on the ethical and moral issues to the observer.”

Evaristti intended the work to act as a social experiment by observing audiences and categorising their choice of engagement into three distinct forms of participation [63]. This involved active (explicit) and passive (implied) ethical responses that reflect current attitudes on human and animal relationships. Evaristti describes these three categories of responses as: “The idiot, who pushes the button; the voyeur, who loves to watch; and the moralist, who will judge the action” [63] (p. 30). Regardless of which category the viewer may be aligned, each has a choice to participate or to not participate, to exit the gallery immediately upon happening upon the artwork or to stay and engage with it. The goldfish in the exhibit have no choice. Perhaps, rather than focusing on “right” or “wrong” actions, this installation explores the role of ethics in contemporary art in order to develop an understanding of moral consciousness being diverse across audience members and in the choice of role that each audience undertakes.

Considering this, Evaristti’s artwork highlights the hierarchal value system between humans and animals by exposing the “levels of worth” of an animal being directly assigned to the value animals have for humans, again echoing the concept of Scala Naturae. In this way, Evaristti (2000) makes the assertion that there is a less sense of moral consciousness in killing a goldfish than other species that are more valued in society. Linking back to Regan’s moral hypothesis the perceived value of an animal should be irrelevant as sentient beings have a basic moral right to life and bodily integrity [65].

These four artists raise ethical concerns when using animals as subject matter within contemporary art. All provided published warnings for viewers that the exhibition content may be confronting, which is in line with art industry protocols. Yet, is a warning sufficient in instances such as these? The strong public reaction to the perceived unethical aspects of the works allows an assessment of what the public believes to be ethical and unethical where animals are involved, and how these responses are mediated by the gallery setting and the artist’s own positioning.

It is important to recognise the difference between the artists discussed in the previous section compared to artists who engage animals in their practice without the requirement to kill or harm them. Contemporary artists deVille and Singer purposefully employ animals in their practice as a strategy to discuss animal rights and offer visibility to the cruel and neglectful treatment of animals within society [66]. By utilising animals in their practice these artists address themes of morality, death and loss [67]. They offer an example of how animals can be ethically included within art. Baker [68] (p. 3) supports the responsible use of animals in art by asserting “the importance of trusting artists to operate with integrity in relation to the animals that figure in their work”.

#### 2.2.6. deVille

Julia deVille is a multidisciplinary artist who works with jewellery and taxidermy. Her practice is distinctive as she uses these skills to transform deceased animals into exquisitely decorated animal installations. These installations include the bodies of cats, cows, birds, mice and other exotic animals who symbolise death and become a reminder of our own mortality. Her practice is influenced aesthetically and thematically by the use of Memento Mori in the period of the 16th–18th centuries; the Victorian era where nature, life, death and most importantly, the humane and kind treatment of all living creatures [69]. deVille’s practice consists of ethical taxidermy, which focuses on preserving life and paying homage to the deceased animal rather than celebrating its death [67]. Ethical taxidermy refers to artists who only source animals in the most humane ways possible and never kill animals for their practice [3]. For some artists this can mean vintage taxidermy mounts or found animals such as “road kill”. However, for deVille this means animals who have died of natural causes. The diversity of approaches emphasises that each artist has to navigate his or her own ethical considerations and limitations within this space when sourcing animals.

deVille’s model of practice is pertinent in critiquing the mistreatment of animals in a way that demonstrates an interweaving of contemporary art, animal rights and activism. This approach challenges her audiences’ morals and advocates for a broader social awareness of animal rights. deVille is able to disrupt the “normal” perception of eating animals by presenting confrontational works such as *Pandora* (2013) [69] which presents a taxidermy lamb on a silver platter with red jewels flowing from it neck. In doing so, her audience is confronted with the idea of death and the importance of all life [69]. By placing deceased animals into a gallery setting, she highlights the strategy of disruption in the repositioning of the way animals are viewed, both in the physical and metaphorical context. deVille [70] states,

“I have firsthand experience of my methods being successful in changing people’s ideas around the way we treat, eat and use animals. As a result of my work and the media coverage it receives, I receive hundreds of emails from people who have become vegan, vegetarian or simply make more ethical decisions around food.”

deVille makes a critical point by noting that even though she utilises animals in her practice she is able to change her audience’s values to perceive animals as beings who are also deserving of moral consideration. While it can be argued that the ethical tensions created by using animals in her practice is an effective form of activism, this strategy is not always supported by the vegan community [71].

#### 2.2.7. Singer

Angela Singer is a vegan artist and animal activist who, like deVille, employs the bodies of deceased animals in her practice to provoke alternative thinking about human relationships with animals. Singer is known for the use of recycled taxidermy installations that expose the unnecessary exploitation of animals and aggression humans inflict on animals. She refers to her technique as “de-taxidermy...a stripping back layer by layer of the animal and the taxidermist’s work” [69] (p. 13). Her practice addresses the unethical approaches of trophy hunting by emphasising wounds on the animals’ bodies through the strategic use of glass beads and wax. *Sore* (2003) [72] presents an old trophy head stripped of its skin that has new “flesh” created with blood red wax. Singer’s practice aims to manipulate reality and taxidermy, allowing the viewer to perceive the animal in a different way. Singer [69] (p. 15) states:

“I wanted to achieve an animal form inspired by the way the stag died but never seen before in nature. Frightening and difficult to look at ‘Sore’ is a powerful work that asks questions about power. Why do humans need to constantly reassure ourselves of our supremacy over other species through the exclusion of that which is not? I discovered that stripping back the skin of the trophy the eye becomes prominent and the work becomes about the gaze; who is the subject watching and who the object? ‘Sore’ appears alive and stares accusingly at us.”

This conceptually and visually layered approach serves as a useful strategy in creating a greater awareness of animal rights. By making the animal look bloody and disfigured, she disrupts the aim of the original piece and exposes the animal’s violent death, presenting a body rather than an object [72]. In this way by seizing and holding the viewer’s attention, the viewer is forced to consider animals that look alive but are in fact dead, presenting the question how and why the animal died.

## 3. Conclusions and Future Perspectives

Given the limited, and yet divergent, Australian and international laws pertaining to animal protection, with little to no guidance given for the ethical treatment of animals used in art, current artistic practice appears to be mediated by individual galleries and their specific governing laws as well as the artists’ own conceptual and philosophical positionings. As demonstrated in this paper, artists’ approaches and views on the matter vary considerably. For example, what one contemporary artist considers the appropriate sourcing of animals for an artwork (an animal who has died by natural cause) is quite different to another artist (slaughtering animals specifically for art), and what one contemporary artist considers is the appropriate inclusion and representation of animals in an artwork (taxidermy installation) differs to another artist (live animal held captive in a gallery). All artists presented within this study justify their actions and their approach to creative expression of content by arguing the artworks are political statements. They are statements designed to provoke the public to think more deeply about animals, our relationships with them, and our contradictory stances on when it is acceptable to kill and consume animals, and when it is not. However, is there a moment when the artistic freedom to utilise animals, whether live or deceased, and treated in whichever way the artist chooses, becomes a case of animal cruelty? Who decides when it is acceptable to directly involve animals without their consent in the name of art made by humans? Further, what mechanisms are put in place in contemporary art to navigate and understand when personal practices trigger, or warrant, public ethical responsibilities and action? This paper is not necessarily advocating for increased censorship, but rather returns to the notion of animal sentience [14] as a yardstick for considering this matter. If, as a society, we accept animal sentience exists, then our perception of animals as intelligent, emotional beings who can reason, make choices, and assess risk, must inform our ethical considerations of the sourcing and inclusion of animals, live and deceased, in our artmaking practices and exhibits.

Within the last few years Australian animal welfare legislation has begun to directly include animal sentience in its Acts (in two States of the country at this stage) foregrounding the importance of this notion and the impact it has on defining what an animal is and how animals are to be ethically treated by humans [13]. During a similar period of time, the Swiss Animal Welfare Act [36] included the notion of preserving the dignity of animals as a framework for ethical treatment during experimentation. As part of this approach the Swiss government generated a protocol of “weighing of interests” [36] to assess whether an animal has been treated ethically and with dignity. In 2017 in the UK, the Animals in Science Committee Harm-Benefit Analysis subgroup reviewed the harm-benefit analysis (HBA) [73] used in the justification of the use of animals in science. The HBA forms part of the ethical review of project license applications involving animals, weighing up the benefits of the research as well as the potential harm to the animals and considering if alternatives could be considered before proceeding. The Queensland Exhibited Animals Act in Australia also sets out specifically how animals are to be treated when on display to the public [33]. Consideration and combination of each of these four foci—sentience, dignity, harm versus benefit, and exhibition—would be a welcome advance in legislation and ethical review to provide contemporary artists with a suitable framework to guide the involvement of animals in their artwork.

The ethical and legal consideration in the use of animals in contemporary art can be further developed by drawing on the very ethical and medical considerations outlined for the public exhibition of plastinated human bodies as per von Hagen’s work. For example, the Federative International Committee for Ethics and Medical Humanities (FICEM) address the ethical concern of displaying human bodies by stating that plastinated human body displays educate from a limited anatomical viewpoint, whereby the body is represented as mechanical material and is depersonalised. A more developed perspective should take on “the body in anatomy not just as a piece of material but also as a (dead) person, thus re-humanising the dead body” [43]. While equivalency has not yet been applied to animal ethics in art, parallels can be made in regard to ideas of animal dignity, and the importance of avoiding an outdated anatomical view being applied to animals as a “specimen” or as mere material for human utilisation. Like human bodies, animal bodies on display should not be presented as a spectacle or be voyeuristic, nor become a morbid curiosity [43].

While the law is lacking, it is recommended that animal ethics discussions are implemented within gallery programming as a means of capturing the spectrum of approaches to the use of animals in art, and the varying complexities regarding animal ethics in art practice, as a means of avoiding what could be considered unacceptable uses of animals within exhibitions. Galleries can also record public responses to the works to generate data to further contribute to the improvement of animal ethics-based laws, policies, and art practices. In order to assist artists and curators who are working with live or deceased animals, the creation of a community of practice informed by the modelling provided by the ACT Animal Welfare Act, the Swiss Animal Welfare Act, the Queensland Exhibited Animals Act, the HBA, and the FICEM will serve as a support network to share potential best practices in ethically sourcing animals would benefit the development and greater consideration of animal ethics in art. Such a community of practice would also enable the opportunity to problem-solve specific complexities regarding the use of animals in works, which can then further inform legislation to ensure animal welfare is regulated in this specific field. In this way, the community of practice is analogous to local ethics committees who review the use of animals in science. Finally, interdisciplinary engagement in the form of papers such as this, along with professional seminars and forums that integrate the arts and sciences in addressing the discourse of animal ethics, may deepen the understanding of some of the key issues addressed through a multi-faceted lens to influence both policy makers and the artistic community across local and global contexts.

## Data Availability

Not applicable.
